# Statement of Retraction: Protective effect of vasostatin-1 plasmid-like nanoparticles on aortic aneurysm and its mechanism

**DOI:** 10.1080/21655979.2024.2299573

**Published:** 2024-02-20

We, the Editors and Publisher of the journal *Bioengineered*, have retracted the following article:

Pingshan Wang, Wei Wang, Xingxing Peng, Fugui Ruan & Shiyao Yang (2022) Protective effect of vasostatin-1 plasmid-like nanoparticles on aortic aneurysm and its mechanism, Bioengineered, 13:1, 544-559, DOI: 10.1080/21655979.2021.2009978.

Since publication, significant concerns have been raised about the integrity of the data and reported results in the article. When approached for an explanation, the authors did not provide their original data or any necessary supporting information. As verifying the validity of published work is core to the integrity of the scholarly record, we are therefore retracting the article. All authors listed in this publication have been informed.

We have been informed in our decision-making by our editorial policies and the COPE guidelines.

The retracted article will remain online to maintain the scholarly record, but it will be digitally watermarked on each page as ‘Retracted’.

